# Neutralization of non-vaccine human papillomavirus pseudoviruses from the A7 and A9 species groups by bivalent HPV vaccine sera

**DOI:** 10.1016/j.vaccine.2011.09.021

**Published:** 2011-11-03

**Authors:** Eve Draper, Sara L. Bissett, Rebecca Howell-Jones, Debbie Edwards, Graham Munslow, Kate Soldan, Simon Beddows

**Affiliations:** a Virus Reference Department, Health Protection Agency, London NW9 5EQ, UK; b HIV/STI Department, Health Protection Agency, London NW9 5EQ, UK; c Bolton NHS, St Peters House, Bolton BL1 1PP, UK

**Keywords:** Human papillomavirus, Vaccine, Antibody

## Abstract

The majority of cervical cancers are associated with infection by one or more Human Papillomavirus (HPV) types from just two distinct Alpha-Papillomavirus species groups, A7 and A9. The extent to which the current HPV16/18 vaccines will protect against other genetically related HPV types is of interest to inform vaccine implementation, cervical disease surveillance and the development of second generation HPV vaccines. The aim of this study was to determine the frequency and titer of neutralizing antibodies against a range of A7 (18, 39, 45, 59, 68) and A9 (16, 31, 33, 35, 52, 58) HPV types using sera from individuals immunized with the bivalent HPV vaccine within the school-based, UK national HPV immunization programme. Serum samples were collected from 69 girls aged 13–14 years, a median 5.9 months (inter-quartile range, IQR, 5.7–6.0) after their third vaccine dose. Cross-neutralizing antibodies against HPV31, HPV33, HPV35 and HPV45 were common and strongly associated with the titer for the related vaccine-type, but were considerably lower (<1%) than their related vaccine type-specific response. The low prevalence of these HPV types in the population and the ages within the study cohort suggest these responses are due to vaccination. It is unclear whether such low levels of neutralizing antibodies would be sufficient to protect at the site of infection in the absence of other immune effectors but the coincidence with HPV types reported from efficacy studies is intriguing. The utility of neutralizing antibodies as surrogate markers of protection remains to be determined.

## Highlights

► We examined neutralizing antibody in girls immunized with bivalent HPV vaccine. ► Antibody cross-reactivity dependent on response to vaccine types. ► Significant cross-reactivity against HPV31, HPV33, HPV45. ► Cross-reactive levels are low at <1% of vaccine type antibody responses. ► Potential role for cross-reactive antibodies as surrogate of protection.

## 1 Introduction

Virus-like particles (VLP) comprising the major capsid protein (L1) of the Human Papillomavirus (HPV) form the basis of the current HPV vaccines, Cervarix^®^ and Gardasil^®^ [1]. Both vaccines target ‘high-risk’ HPV types 16 and 18, which together are associated with *ca.* 70% of cervical cancers [2,3], and demonstrate almost complete protection against HPV16/18-associated high-grade lesions (cervical intraepithelial neoplasia grade 2+; CIN2+) [4,5].

Peripheral immunization with L1 VLP elicits a systemic, type-specific antibody response that coincides with protective immunity at both cutaneous (Cottontail Rabbit Papillomavirus; CRPV) and mucosal (Canine Oral Papillomavirus; COPV) sites in pre-clinical disease models [6,7]. Moreover, naïve animals can be protected from subsequent challenge by passive transfer of serum or purified immunoglobulin G (IgG) from L1 VLP immunized animals. Although the correlates of protection have not yet been defined [8,9], antibodies are the assumed type-specific immune effectors in humans, wherein protection against HPV infection is thought to be imparted by serum antibodies that transudate to the genital mucosa [10–12].

In addition to HPV types 16 and 18, there are another dozen or so HPV types also associated with cervical disease [2,3,13] and the majority of these belong to the same distinct Alpha-Papillomavirus species groups, A7 (HPV18-related: 39, 45, 59, 68) and A9 (HPV16-related: 31, 33, 35, 52, 58) as the vaccine types [14,15]. Emerging clinical trial data suggest that the current HPV vaccines provide a degree of cross-protection against persistent infection and/or high grade lesions (CIN2+) attributed to some of these non-vaccine HPV types, particularly HPV31, 33 and 45, but probably not 52 and 58 [4,16,17]. These findings appear to coincide with limited pre-clinical data showing that HPV16 and 18 VLP can induce low level neutralizing antibodies against genetically related HPV types in small animals [18,19]. Few published data are available on the frequency or titer of neutralizing antibodies raised in vaccinated humans against closely related, non-vaccine types, HPV31, HPV45, HPV52 and HPV58 [20,21]. A recent study exploring alternative dosing schedules suggested that there was little difference in vaccine-type antibody titers induced by two or three doses of Gardasil^®^ [22]. The potential impact of a reduced dosing schedule on the induction of vaccine-specific, cross-reactive antibodies is unknown.

In this study we have evaluated the propensity for serum from 13 to 14 year old girls immunized with the bivalent vaccine, Cervarix^®^, within the school-based, UK national immunization programme, to cross-neutralize pseudoviruses representing a range of A7 and A9 ‘high risk’ HPV types.

## 2 Materials and methods

### 2.1 Study samples

Anonymized serum samples were collected, following informed consent, from 13 to 14 years old girls approximately six months after completion of a three-dose vaccination schedule with the bivalent HPV vaccine, Cervarix^®^. The vaccines were delivered through the UK's school-based national HPV Immunization Programme within the recommended dosing intervals [23].

Anonymized serum samples from infants (6 months to 4 years old, males and females) participating in a clinical trial where consent had been given for anonymous testing for other vaccine-related antibodies were used to gauge the potential for non-specific assay interference. Environmentally acquired HPV infection was expected to be rare in this group and, for the present purposes, we considered these individuals to represent a true HPV negative population; herein referred to as HPV-naïve.

### 2.2 Pseudovirus neutralization assay

Bicistronic vectors (pXsheLL, where X is the Papillomavirus type from which the codon optimized L1 and L2 genes were derived) representing the HPV types 16, 18, 31, 45, 52, 58 and Bovine Papillomavirus (BPV) were obtained from J.T. Schiller, National Cancer Institute, Bethesda, MD, USA, while p33sheLL and p68sheLL were obtained from H. Faust and J. Dillner, Malmö University Hospital, Malmö, Sweden. Constructs representing HPV35 (p35sheLL), HPV39 (p39sheLL) and HPV59 (p59sheLL) were generated by the insertion of codon optimized genes (Blue Heron, Inc., Bothell, WA, USA) based upon consensus L1 and L2 amino acid sequences into p5shell (http://home.ccr.cancer.gov/lco/default.asp). The consensus sequences were derived from NCBI database sequences (HPV35: M74117, X74477; HPV39: M62849; HPV59: X77858, EU918767) and contemporary sequences from anonymous, HPV-infected cytology samples (HPV35 L1: JN104062–64; HPV35 L2: JN104065–67; HPV39 L1: JN104068–70; HPV39 L2: JN104071–72; HPV59 L1: JN104073–74; HPV59 L2: JN104075–77).

The production of L1L2 pseudovirus stocks was performed as described elsewhere [24] using the alternative protocol (http://home.ccr.cancer.gov/lco/ripcord.htm), developed to reduce the inclusion of excess non-reporter-containing ‘cold capsids’, and by using luciferase (pGL4.51 [*luc2/CMV/Neo*]; Promega, Madison, WI) as the encapsidated reporter. Briefly, 293TT cells were transfected with equal amounts of pXsheLL and pGL4.51 [*luc2/CMV/Neo*] plasmids (Lipofectamine 2000; Invitrogen, Carlsbad, CA) and the encoded proteins expressed for 48 h before the cells were lysed, the capsids matured overnight in the presence of ribonucleases (RNase Cocktail; Applied Biosystems/Ambion, Austin, TX) and the double-clarified supernatant subjected to iodixanol gradient fractionation.

Purified pseudovirus stocks were titrated on 293TT cells in quadruplicate, five-fold serial dilutions and the equivalent of a Tissue Culture Infectious Dose 50% (TCID_50_) was estimated using the Spearman–Karber equation. The average of three such estimations was made for each pseudovirus stock used in this study. Pseudovirus-mediated reporter gene transduction of target cells in both the infectivity and neutralization assays was measured using the Steady-Glo Luciferase Assay Reagent (Promega) and the Glomax Multi Detection System (Promega) according to manufacturer's instructions.

The HPV pseudovirus neutralization assay was performed as originally described [25] with some modification. For the present study, heat-inactivated (56 °C, 30 min) serum samples were initially screened against all pseudoviruses (at a final serum dilution of 1/20 with pseudovirus) and any serum that demonstrated ≥80% reduction in the luciferase signal (RLU) relative to the pseudovirus and cell only controls was subsequently titrated and an 80% reciprocal neutralization titer estimated by interpolation.

It was important to ascertain the appropriate input level of pseudovirus for the neutralization assays. A plot of input TCID_50_ against output luciferase signal (RLU) demonstrated that 300 TCID_50_ was within the linear range of the assay for all A7, A9 and BPV pseudoviruses and a median 3.35 (inter-quartile range, IQR, 3.14–3.56; *n* = 4–9 tests per HPV type) Log_10_ fold over the background level of the assay; linear regression, *r*^2^ = 0.908 (IQR, 0.862–0.933) [26]. The median level of L1 protein at this level of input, determined for the A9 pseuodviruses, was 0.04 (IQR, 0.02–0.1) ng/mL. This level is at least an order of magnitude lower than that reported by Pastrana et al. [25], as expected, due to the removal of ‘cold capsids’ using the alternative protocol. However, a comparison of HPV16 and HPV31 neutralization titers derived using 30, 300 and 3000 input TCID_50_, spanning *ca.* 4 Log_10_ range of L1 protein and *ca.* 2 Log_10_ difference in particle to infectivity ratios between the standard and alternative protocol-produced stocks were not significantly different (Wilcoxon paired signed rank test and analysis of trend; *p* > 0.05). Thus, 300 TCID_50_ was deemed an appropriate pseudovirus input and used for all subsequent neutralization assays.

Inter-assay reproducibility of neutralizing antibody titers was demonstrated by including in every experiment a vaccinee serum pool control, comprising study sera selected following an initial neutralization screen against HPV16, HPV18, HPV31, HPV45, HPV52 and HPV58. Median (IQR; *n*) neutralization titers were as follows: HPV16 65,564 (59,607–82,880; *30*); HPV31 449 (322–499; *26*); HPV33 62 (57–75; *25*); HPV35 21 (17–24; *26*); HPV52 43 (33–59; *25*); HPV58 413 (370–507; *25*); HPV18 17,632 (14,660–21,593; *14*); HPV39 <20 (N/A; *6*); HPV45 70 (43–89; *9*); HPV59 <20 (N/A; *7*); HPV68 <20 (N/A; *7*); BPV <20 (N/A; *19*). As HPV39, 59, 68 and BPV were not neutralized by this control serum pool, neutralization tests using these pseudoviruses were repeated against all study sera to confirm the lack of activity and included Heparin (H-4784; Sigma, UK) as a positive inhibitor control. All A7, A9 and BPV pseudoviruses were sensitive to heparin with a median 80% inhibition concentration of 14.3 (IQR, 3.2–21.9) μg/mL [27–29].

A small panel of nine sera samples was also retested at the end of the study against six pseudoviruses HPV16, 31, 33, 35, 52 and 58 (*n* = 54; linear regression, *r*^2^ = 0.983; Wilcoxon Paired Signed Rank Test for differences between groups, *p* = 0.629).

### 2.3 Data analysis

2-tailed Fisher's exact test and two sample Wilcoxon rank-sum (Mann–Whitney) test were used to compare proportions of individuals with positive neutralizing antibody and antibody titers of vaccinees versus HPV-naïve individuals, respectively. Spearman's and Kendall's rank correlations and Pearson's product-moment correlation were used to compare the neutralizing antibody titers against non-vaccine types and the corresponding vaccine type within a species group. Wilcoxon paired signed rank test was used to determine whether the range of neutralizing antibody titers against two HPV types were different. Kruskal–Wallis equality-of-populations rank test and the test for trend across ordered groups (trend analysis) were used to assess the difference between non-vaccine type neutralization data ordered into tertiles based upon neutralizing antibody titers against the vaccine type. All tests were performed using the statistical package, Stata 10.1 (StataCorp, College Station, TX).

## 3 Results

Sixty-nine serum samples were collected a median 5.9 (IQR 5.7–6.0) months after receiving a third dose of the Cervarix^®^ vaccine.

As expected, all (*n* = 69, 100%) individuals generated high titer neutralizing antibodies against HPV16 and HPV18 following vaccination (Table 1), with HPV16 titers a median 3.5 (IQR, 1.7–5.8) fold higher than the corresponding HPV18 titers (Wilcoxon paired signed rank test; *p* < 0.001). Sera capable of neutralizing non-vaccine A9 HPV types were commonly found among this group of vaccinees (ranging from 15% to 87% of individuals, depending on the HPV type) with neutralization detected most frequently for HPV31, followed by (in order) 33, 52, 35, and 58. Sera capable of neutralizing non-vaccine HPV types within the A7 species group were fewer and almost completely restricted to reactivity against HPV45. No inhibition of the control BPV pseudovirus was seen using these vaccine sera.

### 3.1 Specificity of the pseudovirus neutralization assay for vaccine-induced responses

Little or no non-specific inhibition of pseudovirus entry was seen using the HPV-naïve sera resulting in an apparent assay specificity of 99–100% (Table 1). The exception was pseudovirus HPV52 which was inhibited by four sera, albeit to low titer, resulting in an apparent specificity of 95% (95% CI, 90–100) for this HPV type. No inhibition of the control BPV pseudovirus was seen using these HPV-naïve sera.

### 3.2 Association of vaccine-type and non-vaccine type responses

Significant associations were found between the neutralizing antibody titers observed against HPV31, 33, 35, 45, 52 and 58 and the titers observed against their related vaccine-type (Spearman's and Kendall's rank correlation, *p* < 0.005; data not shown). However, using the more stringent Pearson's product-moment correlation coefficient only HPV31 (*r* = 0.855; *p* < 0.001), HPV33 (*r* = 0.523; *p* < 0.001), HPV35 (*r* = 0.269; *p* = 0.026) and HPV45 (*r* = 0.485; *p* < 0.001) gave significant associations with their respective type-specific titers. As expected [12], a significant correlation was found between the neutralizing antibody titers for HPV16 and HPV18 (Spearman's *rho* = 0.673; *p* < 0.001; Pearson's *r* = 0.657; *p* < 0.001).

The relationship between vaccine-type and non-vaccine type neutralization was further investigated by subdivision of the sera into tertiles based on the vaccine-type titers for each species group (HPV16 tertiles for A9 types and HPV18 tertiles for A7 types). For HPV types 31, 33, 35, 45 and 58 the percentage of individuals with a positive, non-vaccine type neutralization titer increased with each tertile of vaccine-type titer (Table 2). For example, 70% of individuals in the low HPV16 tertile had a positive neutralizing antibody titer against HPV31, rising to 91% for the middle and 100% for individuals in the high HPV16 tertile. This was not the case for HPV52, however, which demonstrated no increase in positivity between the middle and high tertiles. The number of non-vaccine types neutralized per serum increased with type-specific tertile such that the median number of non-vaccine types neutralized by sera in the lowest HPV16 tertile was 1.0 (IQR, 0.5–1.5) compared with 2.0 (2.0–2.5) and 3.0 (IQR, 1.5–4.0) for the middle and high tertiles, respectively.

**Fig. 1:**
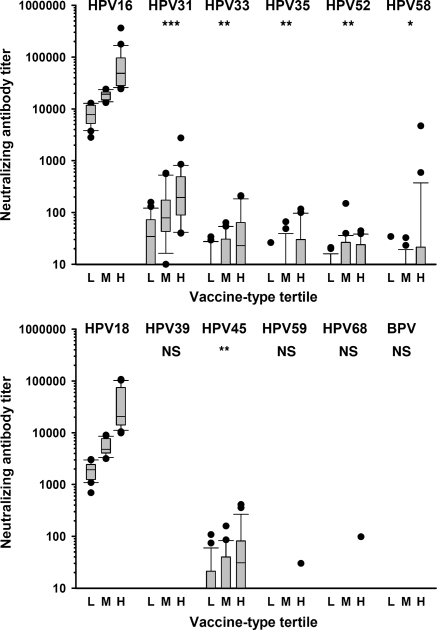
Cross-neutralizing antibody titers are related to vaccine-type neutralizing antibody titers for A9 HPV types (top panel) and A7 HPV types (bottom panel). Neutralizing antibody data for non-vaccine types segregated according to Low (L), Middle (M) and High (H) vaccine-type tertiles. Plot shows box (median, IQR), whisker (±1.5 IQR) and outliers (>1.5 IQR). *p* values represent association by trend across tertiles: *p < 0.05; **p < 0.01; ***p < 0.001; ^NS^*p* > 0.05.

Neutralizing antibody titers against non-vaccine types HPV31, 33, 35, 45, 52 and 58 increased in association with increasing vaccine-type tertiles (Table 2 and Fig. 1). For example, for HPV31, the median (IQR) titer was 34 (10–71) for the low HPV16 tertile, rising to 78 (47–169) for the middle and 195 (92–490) for the high HPV16 tertile. Significant associations were found between cross-neutralizing titers for non-vaccine types and vaccine-type tertile for HPV31, 33, 35, 45, 52 and 58) when assessed by the Kruskal–Wallis test (data not shown) or the test for trend across ordered groups (Table 2 and Fig. 1). As expected, HPV18 neutralizing antibody titers were significantly associated with increasing HPV16 tertiles (trend analysis and Kruskal–Wallis test; *p* < 0.001).

### 3.3 Relative magnitude of cross-neutralizing antibody responses

Cross-neutralization titers were overall very low, being <1% of the respective type-specific, HPV16 or HPV18 titer: for example, HPV31 (median 0.49% [IQR 0.24–1.02%]), HPV33 (0.13% [0.09–0.24%]) and HPV45 (0.50% [0.18–1.02%]). In contrast to the increase across the vaccine-type tertiles of the percentage of individuals with, and levels of, cross-neutralizing titers (Table 2), the relative magnitude of non-vaccine to vaccine titers decreased across the tertiles. For example for HPV31, the median (IQR) percentage of type-specific titer was 0.69% (0.47–1.08%) for the low HPV16 tertile, falling to 0.49% (0.25–1.07%) for the middle and 0.29% (0.17–0.77%) for the high HPV16 tertile (trend analysis; *p* = 0.018).

## 4 Discussion

In this study we have attempted to estimate the propensity for serum taken from 13 to 14 year old girls recently vaccinated with the bivalent HPV vaccine to neutralize pseudoviruses representing genetically related, non-vaccine HPV types within the A9 and A7 species groups. Neutralizing antibodies against non-vaccine A9 HPV types were commonly detected within this study group, with antibodies against HPV31 and HPV33 being the most frequently detected and of the highest titer. The only A7 non-vaccine HPV type for which a significant neutralizing antibody response was found was HPV45.

Neutralizing antibody titers against HPV31, 33, 35, 45 (and to a lesser extent HPV52 and 58) were significantly associated with their related vaccine-type antibody titers, suggesting that the generation of cross-neutralizing antibodies is at least coincident with the host immune response to vaccination. No significant neutralization was seen against the A7 HPV types 39, 59 and 68 or the control BPV. It is noteworthy perhaps that the individuals with a positive neutralizing antibody score against either HPV59 or HPV68 were also in the highest tertile of vaccine-type HPV18 neutralizing antibody titers, suggesting that responses against these types, although not significant overall and a rare occurrence (<5% of vaccinees), may indeed be vaccine-related. The fewer number of samples positive for neutralizing antibodies against non-vaccine HPV types from the A7 species group, being almost exclusively directed against HPV45, than from the A9 species group, is likely to be related to the lower (3.5 fold) titers generated against the vaccine-type HPV18 compared to HPV16, which appears to be a common finding for the HPV vaccines [12,30–32]. Cross-neutralizing antibody titers were substantially lower (<1%) than vaccine-type titers and the gap between these two measures widened with increasing vaccine-type titer. These observations suggest that individuals who elicit the highest antibody responses against vaccine types generate the highest absolute levels of cross-reactive antibodies but the lowest cross-reactive responses as a function of their vaccine-type responses, perhaps reflecting the immunodominance of the type-specific neutralizing epitope(s) relative to the cross-reactive epitope(s).

A recent study [20] provided evidence for significant cross-neutralization of HPV31 and HPV45 (but not HPV52 and HPV58) pseudovirions using sera taken from 18 to 25 year old women six months after immunization with the bivalent HPV vaccine as part of a clinical trial in Costa Rica [33]. Antibody cross-reactivity against HPV45 has also been reported for the quadrivalent HPV vaccine, Gardasil^®^ [21]. The discrepant observations concerning HPV52 and HPV58 between this study and the analysis by Kemp et al. [20] may be due to differences in the ages of the study participants, a parameter known to have an impact on HPV vaccine immunogenicity [31].

We have expanded the currently available panel of HPV L1L2 pseudoviruses to represent all those HPV types within the vaccine-related A9 (16, 31, 33, 35, 52, and 58) and A7 (18, 39, 45, 59, 68) species groups that have been considered by the International Agency for Research on Cancer to be at least ‘probably carcinogenic to humans’ [13]. We are not aware of any published data on the measurement of cross-neutralizing antibodies elicited against the closely related, non-vaccine types HPV33, HPV35, HPV39, HPV59 and HPV68 by either HPV vaccine.

We did not have pre-vaccine sera, or sera from unvaccinated 13–14 year old girls, with which to gauge background levels of naturally induced HPV antibodies and non-specific assay interference. However, the sera included in our study were from young girls close to the target age (12 years) for routine HPV immunization in the UK and within an age-group where the level of naturally induced antibodies was expected to be very low [34]. This leads us to believe that significant confounding due to prior infection with, and immune response to, non-vaccine types to be highly unlikely. Our assessment of non-specific interference using sera from HPV-naïve infants resulted in a pseudovirus neutralization assay specificity of around 99–100%.

As the sera used for this study were collected within six months of the third vaccine dose and given the apparent improved immunogenicity within this age group [31], the titers of cross-neutralizing antibodies reported here are likely to represent peak levels. Type-specific neutralizing antibodies appear to wane quite quickly following vaccination to plateau several fold lower than their peak level [35] and this is likely to be true also for cross-neutralizing antibodies. We did not have repeat samples or a sufficient range in collection times to assess changes in neutralizing antibody titers over time.

The detection of cross-neutralizing antibodies in vaccine sera *per se* does not, of course, provide sufficient evidence for antibodies being mechanistically associated with cross-protection. Furthermore, type-specific antibody titers in genital secretions are orders of magnitude lower than those found in the periphery [12] and it is unclear whether these very low levels of cross-neutralizing antibodies found in the periphery would be sufficient to protect at the site of infection in the absence of other immune effectors [36,37]. However, the coincidence of the rank order of HPV types recognized by vaccinee sera in this and other studies [20] and the apparent hierarchy of protected HPV types suggested from efficacy studies [4,16,17] is intriguing. Defining the mechanism(s) of cross-protection will be important to monitor vaccine effectiveness on both a population and individual level.

These data may be helpful to parameterize epidemiological models to predict the impact of the current HPV vaccines on the population and to inform the development of second generation HPV vaccines.

## Ethical approvals

This study was given a favorable ethical opinion by the Tameside & Glossop Local Research Ethics Committee, Manchester, UK (REC reference number 09/H1013/33).

## Figures and Tables

**Table 1:** Cross-neutralization of A9 and A7 pseudoviruses by bivalent vaccine or HPV-naïve sera.

Clade	PsV	Vaccinees			HPV-naïve			*p* value^b^
		Median (IQR) titer^a^			Median (IQR) titer			
		*N* (%)	All	Positives	*N* (%)	All	Positives	
A9	16	69 (100)	19,258 (11,730–28,132)	19,258 (11,730–28,132)	0 (0.0)	10 (10–10)	N/A	<0.001
	31	60 (87.0)	78 (40–173)	96 (50–203)	1 (1.0)	10 (10–10)	23 (23–23)	<0.001
	33	29 (42.0)	10 (10–27)	29 (25–54)	0 (0.0)	10 (10–10)	N/A	<0.001
	35	15 (21.7)	10 (10–10)	30 (25–67)	1 (1.0)	10 (10–10)	23 (23–23)	<0.001
	52	22 (31.9)	10 (10–21)	25 (21–30)	4 (3.8)	10 (10–10)	27 (26–27)	<0.001
	58	10 (14.5)	10 (10–10)	33 (25–45)	0 (0.0)	10 (10–10)	N/A	<0.001

A7	18	69 (100)	4775 (2442–14,149)	4775 (2442–14,149)	1 (1.4)	10 (10–10)	25 (25–25)	<0.001
	39	0 (0.0)	10 (10–10)	N/A	0 (0.0)	10 (10–10)	N/A	N/A
	45	29 (42.0)	10 (10–45)	48 (31–85)	0 (0.0)	10 (10–10)	N/A	<0.001
	59	1 (1.4)	10 (10–10)	30 (30–30)	1 (1.4)	10 (10–10)	23 (23–23)	0.976
	68	1 (1.4)	10 (10–10)	98 (98–98)	0 (0.0)	10 (10–10)	N/A	0.310
	BPV	0 (0.0)	10 (10–10)	N/A	0 (0.0)	10 (10–10)	N/A	N/A

N/A, not applicable.

a Number of samples with neutralizing antibody titers ≥ 20 and, in parentheses, percentage of total vaccinee sera (*n* = 69) and HPV-naïve sera (A9, *n* = 104; A7, *n* = 71; and BPV, *n* = 98) tested. Median (IQR, interquartile range) neutralizing antibody titers of all responses, by assigning an arbitrary value of 10 to titers < 20, or only those considered positive (neutralizing antibody titers ≥ 20).

b Two-sample Wilcoxon rank-sum (Mann–Whitney) test for comparison between all neutralization titers using vaccinee and vaccine-naïve sera. Similar associations were obtained when examining the proportion of sera positive for neutralizing antibody against HPV16, 31, 33, 35, 52, 58, 18 and 45 (*p* < 0.001), HPV59 (1.000) and 68 (0.493), using 2-tailed Fishers Exact test.

**Table 2:** Comparison of cross-neutralizing antibody titers with type-specific titers.

Clade	HPV	Tertile^a^	Titer^b^		*N* (%)^c^	% of type-specific titer^d^	
			Median (IQR)	*p* value		Median (IQR)	*p* value
A9	31	Low	34 (10–71)		16 (70)	0.69 (0.47–1.08)	
		Middle	78 (47–169)		21 (91)	0.49 (0.25–1.07)	
		High	195 (92–490)	**<0.001**	23 (100)	0.29 (0.17–0.77)	**0.018**

	33	Low	10 (10–10)		4 (17)	0.26 (0.23–0.29)	
		Middle	10 (10–31)		11 (48)	0.18 (0.12–0.25)	
		High	23 (10–46)	**0.003**	14 (61)	0.07 (0.04–0.12)	**0.002**

	35	Low	10 (10–10)		1 (4)	0.22 (0.22–0.22)	
		Middle	10 (10–10)		5 (22)	0.12 (0.11–0.24)	
		High	10 (10–29)	**0.003**	9 (39)	0.10 (0.03–0.11)	**0.023**

	52	Low	10 (10–10)		2 (9)	0.30 (0.24–0.35)	
		Middle	10 (10–26)		10 (43)	0.13 (0.12–0.16)	
		High	10 (10–24)	**0.009**	10 (43)	0.04 (0.03–0.05)	<**0.001**

	58	Low	10 (10–10)		1 (4)	0.27 (0.27–0.27)	
		Middle	10 (10–10)		2 (9)	0.16 (0.14–0.18)	
		High	10 (10–21)	**0.013**	7 (30)	0.03 (0.02–1.17)	0.269

A7	39	Low	10 (10–10)		0 (0)	N/A	
		Middle	10 (10–10)		0 (0)	N/A	
		High	10 (10–10)	N/A	0 (0)	N/A	N/A

	45	Low	10 (10–16)		6 (26)	2.02 (1.14–3.47)	
		Middle	10 (10–36)		8 (35)	1.00 (0.75–1.55)	
		High	31 (10–76)	**0.003**	15 (65)	0.18 (0.13–0.35)	<**0.001**

	59	Low	10 (10–10)		0 (0)	N/A	
		Middle	10 (10–10)		0 (0)	N/A	
		High	10 (10–10)	0.221	1 (4)	N/A	N/A

	68	Low	10 (10–10)		0 (0)	N/A	
		Middle	10 (10–10)		0 (0)	N/A	
		High	10 (10–10)	0.221	1 (4)	N/A	N/A

N/A, not applicable.

a Tertiles based on equal distribution (*n* = 23 sera per tertile) of type-specific neutralization titers for HPV16 (Low: median 7763 [IQR, 5305–11,438]; Middle: 19,258 [15,788–20,949]; High: 48,687 [29,016–95,043]) and HPV18 (Low: 1929 [1250–2399]; Middle 4775 [4104–7209]; High: 20,582 [15,177–68,027]).

b Median (IQR) neutralization titer for all samples in each tertile (*n* = 23), substituting 10 for a neutralization titer of <20. *p* value generated by trend analysis with significant associations highlighted in bold type.

c Number of sera positive for neutralizing antibody against indicated HPV type and, in parentheses, percentage of total (*n* = 23) sera in each tertile.

d Median (IQR) percentage of type-specific neutralization titer using samples positive for cross-neutralizing antibodies. *p* value generated by trend analysis with significant associations highlighted in bold type.
